# Anemia and Iron Deficiency in Pregnancy

**DOI:** 10.1155/2012/241869

**Published:** 2012-08-28

**Authors:** Alexander Krafft, Laura Murray-Kolb, Nils Milman

**Affiliations:** ^1^Division of Obstetrics, Department Obstetrics and Gynecology, University Hospital Zurich, Frauenklinikstrasse 10, 8091 Zurich, Switzerland; ^2^Department of Nutritional Sciences, The Pennsylvania State University, 219 Chandlee Lab, University Park, PA 16802, USA; ^3^Department of Gynecology & Obstetrics, Næstved Hospital, Ringstedgade 61, 4700 Næstved, Denmark

Iron deficiency is still the world's most common nutritional deficiency, and generally, iron deficiency anemia is the most prevalent form of anemia. The major risk groups for iron deficiency include women of childbearing age, pregnant women, and lactating postpartum women. There exist plenty of studies attending and reconfirming this important issue. To combat this problem, different food iron fortification programmes have been implemented in some regions, iron supplementation guidelines have been elaborated in some countries, and iron therapy schemes involving both oral and intravenous administration have been proposed. But despite these efforts and the recommendations by the World Health Organization, the problem concerning iron deficiency and anemia is still unsolved in most parts of the world. Even in many industrialized countries, the topic of iron and pregnancy often stays unattended and there is a lack of consensus concerning guidelines in different countries.

Other disorders, such as hemoglobinopathies, also contribute to the high prevalence of anemia worldwide. The prevalence of anemia is influenced by a variety of deficiencies (e.g., folate, vitamin B12, vitamin A, vitamin D, and vitamin C), infectious diseases, parasitic infestations, inflammatory diseases as well as malnutrition. 

As data for iron are quite profound, these are often scarce if we look on other vitamins and micronutrients, except for folic acid. As we know, periconceptional supplementation with folic acid has the potential to reduce the incidence of neural tube defects (e.g., spina bifida) dramatically. Unfortunately, folic acid prophylaxis is not commonly used for several reasons.

By this special issue, our intention was to broaden the perspective on anemia in pregnancy and propose ways to solve the problems. We have chosen three articles concerning the prevention/treatment of iron deficiency anemia in pregnancy/postpartum including papers from Australia, Nigeria, and Denmark. Another Danish study focuses on the prevention of postpartum anemia through hemostatic sealing of the placental bed at caesarean section, thereby reducing perioperative blood losses. A paper from France and Lebanon evaluates the relationship between periconceptional folate deficiency and neural tube defects. A Chinese study addresses the supposed relationship between iron deficiency and postpartum depression. Together, these papers illustrate some of the many facets of the complexity of the prevention and treatment of anemia in pregnant and postpartum women.

*Alexander Krafft* *Alexander Krafft* *Laura Murray-Kolb* *Laura Murray-Kolb* *Nils Milman* *Nils Milman*

